# LwaCas13a indiscriminately targets the human transcriptome

**DOI:** 10.12688/wellcomeopenres.23656.2

**Published:** 2025-11-22

**Authors:** Aguilar-Martinez E., Tonthat G., Antony Adamson, Kurinna S.

**Affiliations:** 1 Faculty of Biology. Medicine and Health, University of Manchester, Manchester, Greater Manchester, M13 9PT, UK

**Keywords:** LwaCas13a; down-regulation; off-target; trans-collateral activity; RNA

## Abstract

**Background:**

Clustered Regularly Interspaced Short Palindromic Repeats (CRISPR)-associated protein 13a (Cas13a) has been described as a superior tool to short-interfering RNAs (siRNAs) for specific gene silencing. Cas13 targets RNAs through Watson-Crick binding of the target CRISPR RNA (crRNA) and activation of nuclease activity. In bacteria, once *Leptotrichia wadei* Cas13a (LwCas13a) has cut its specific target, the trans-collateral activity of the protein degrades any single-stranded RNA present in the cell independent of its sequence or homology to the crRNA. This transcollateral activity has been reported to be absent in mammalian cells. Therefore, in this study, we aimed to downregulate mRNAs expression in mammalian cells (HaCaT and HEK293T) using LwaCas13a.

**Methods:**

We developed a doxycycline-inducible system to express LwaCas13a in HEK293T cells. The off-target activity of LwaCas13a in HEK293T cells was analyzed using RNA-seq.

**Results:**

In this study, we observed that activation of LwaCas13a in HEK293T cells led to non-specific targeting of RNAs, which caused cell toxicity and death.

**Conclusion:**

This study provides evidence of the off-target activity of LwaCas13a in HEK293T cells, making it an unsuitable tool for the specific downregulation of RNAs.

## Plain Language summary

Bacteria have developed an immune system called CRISPR, which can specifically recognize and destroy foreign DNA or RNA. When CRISPR-associated proteins, such as Cas12 or Cas13, are activated, they exhibit not only targeted activity but also non-specific activity, which can degrade any DNA or RNA within the bacteria. The CRISPR system, which consists of proteins and guide RNA molecules, has been adapted to function in mammalian cells, where it is used for genome editing or gene expression regulation. Previous studies have suggested that the non-specific activity observed in bacteria does not occur in mammalian cells. However, in our study, we used CRISPR to downregulate RNAs in mammalian cells and discovered unexpected non-specific activity of CRISPR proteins. This unintended activity results in cell toxicity and cell death.

## Introduction

Clustered regularly interspaced short palindromic repeat (CRISPR)-associated proteins (Cas) are components of the prokaryotic adaptive immune system ( Barrangou *et al.*, 2007). Cas proteins bind to and cleave foreign nucleic acids. Class I proteins include those that work as part of a complex, whereas class II proteins do not require another Cas protein to work. Cas proteins can bind to and cleave double-stranded DNA (Cas9 and Cas12) or single-stranded RNA (Cas13). Target specificity is based on the ability of Cas proteins to bind an RNA molecule, CRISPR RNA (crRNA or gRNA), which is complementary to the target nucleic acid ( Ishino *et al.*, 2018). For Cas13, the crRNA is a small RNA molecule formed by a conserved sequence, the direct repeat, which is bound by the protein, and a 28-nucleotide long sequence, the spacer, complementary to the target RNA. Single or double mismatches in the crRNA-target duplex reduce the knockdown activity of Cas13 ( Abudayyeh *et al.*, 2017). In prokaryotic cells, once Cas12 and Cas13 have found and cleaved their specific targets, trans-collateral activity is turned on, resulting in a crRNA-independent, unspecific cleavage of any single stranded DNA (Cas12) or single stranded RNA (Cas13) present in the cell. This prevents cell growth, and as a consequence, the phage infection cycle ceases ( Ishino *et al.*, 2018; Meeske *et al.*, 2019).

Cas proteins have been used in mammalian cells to edit the genome (Cas9) or regulate gene expression at the RNA level ( Hillary & Ceasar, 2023). Since their discovery, several Cas13 proteins that bind and cleave RNA have been described ( Devillars *et al.*, 2024). The first reports of using Cas13 to downregulate mammalian messenger RNA described Cas13 as a superior alternative to small interfering RNAs (siRNAs), with nearly no off-target effects. In addition, reports have emphasized that the trans-collateral activity of Cas13, seen in prokaryotic cells, was not present in mammalian cells ( Abudayyeh *et al.*, 2017). Therefore, we used *Leptotrichia wadei* Cas13a (LwaCas13a) to knockdown specific mRNAs in mammalian cells. However, contrary to expectations, the expression and activation of LwaCas13a resulted in cell toxicity and the degradation of collateral cellular mRNAs.

## Methods

### Cell culture

HEK293T and HaCaT cells were grown in DMEM-high glucose (Sigma, D5796) supplemented with 10% heat inactivated foetal bovine serum, (Gibco, 10500064). Cells were cultured at 37° C, 5% CO ^2^ in a humidified incubator. The cells were passaged every 2–3 days using 0.05% trypsin-EDTA (Thermo Fisher Scientific, 253000). LwaCas13a expression was induced by adding doxycycline (Sigma D9891) to a final concentration of 1 μg/ml to the cells grown in media supplemented with TET-free foetal bovine serum (Takara, 631107) for 48 h. HEK293T cells were transfected using Polyfect (Qiagen, 301105), following the manufacturer’s protocol. Puromycin (Gibco, A1113803) was used to select cells expressing LwaCas13a-Puro at a concentration of 1 μg/ml. *KRAS* gRNA expression was induced by adding doxycycline to a final concentration from 50-1,000 μg/ml.

### Plasmid constructs, gRNAs and gDNA PCR

All PCR fragments were generated using Q5® high-fidelity DNA polymerase (NEB, M0491S). The LV-Cas13a-T2A-Puro plasmid was created using HiFi assembly (NEB, E5520S). Fragments, 1.Cas13a-HA amplified using primers set Cas13a-Gib-5’ (agaagttgtggggaggggtcggcaattgaaccggtggtacctaggtcttgaaaggagtgggaattgg) and Cas13a-Gib-3’ (agcagacttcctctgccctcggcatagtcggggacatcatatgggtatgcataatcaggcacgtcgtagggataagcgtaatctggaacatcgtatgggtatttcttcttcttagcctgtccagccttctttgtggcggcaggtcgcttgctacctcctcctccgcttcc) and template addgene plasmid #91902 (pC014-LwaCas13a-msGFP) and 2.T2A-Puro amplified by PCR using the primers set T2A-Puro-Gib-5’ (atgatgtccccgactatgccgagggcagaggaagtctgctaac) and SKU02b-Puro-R (ggccgggctagcttagaattctcaggc), were ligated in to the KpnI/NheI cut pLV-MCS plasmid. The pLV-MCS plasmid was created by replacing AgeI-BsmBI of Addgene plasmid #110821with a 94 bp long sequence with multiple restriction enzyme sites. LV-Cas13a-Puro-TetR was cloned by HiFi assembly inserting the Ubc promoter-TetR PCR product (primers, UbC-NheI-5' gccggcgctagcggcctccgcgccgggttttgg and TetR-NotI-3' ggccgggcggccgcttaagagccagactcacatttcag) from Addgene plasmid 70183 into the NheI/NotI site of LV-Cas13a-Puro plasmid. Tet-LV-gRNA-13a-GFP was cloned by HiFi assembly (NEB) of a gblock (IDT) containing U6-promoter-Tet-operator-LwaCas13a-DR into the NdeI-EcoRI sites of pLKRO5-GFP. *KRAS* gRNA (caaggcactcttgcctacgccaccagct) was cloned into the BsmbI sites of Tet-LV-gRNA-13a-GFP following ( Ran *et al.*, 2013).

The LV-mCherry-T2A-Cas13a plasmid was created using HiFi assembly (NEB, E5520S). Four fragments, corresponding to 1. mCherry, amplified using the primer set mCherry13-F (ccctcgtaaattaattatggctagcgtttaaacgggccctctagagccaccatggtgagcaagggc) and mCherry13-R (gtggcggcaggtcgcttcattgggccaggattctcctcg), 2.LwaCas13a, amplified by PCR from Addgene plasmid #91902 (pC014-LwaCas13a-msGFP) using the forward primer mCherr-Cas13-F (tcgaggagaatcctggcccaatgaagcgacctgccgc) and reverse primer mCherr-Cas13-R (gtggcggcaggtcgcttcattgggccaggattctcctcg); 3. WPRE was amplified using the primers WPRE-F (ccctcaatccagcggaccttccttcccgcggcctgctgccggctctgcggcctcttccgc) and W-CMV-R (cttgtccagccggctcattcggtctagcggatctgacggt), and 4. CMV enhancer-TetON3G amplified using the forward TetON-F (accgtcagatccgctagaccgaatgagccggctggacaag) and reverse TetON-R (gatctacagctgccttgtaagtcattggtcttaaaggtaccctatccgggcagcatgtccagg) primers was ligated into the KpnI-NaeI-linearized HB4-B6 plasmid.

LV-gRNA-13a-GFP was created by replacing the NdeI-EcoRI sequence of pLKO5.sgRNA.EFS.GFP with a U6-promoter-LwaCas13a scaffold flanked by the BsmBI sites. LV-sgRNA-13a-GFP-Neo and LV-sgRNA-13a-GFP-MALAT1 containing gRNA targeting *Neomycin* (cttgacaaaaagaaccgggcgcc) or *MALAT1* (caaaactccaagaactagcacctgcaga) were cloned following ( Ran *et al.*, 2013). 

Genomic DNA was extracted using the PureLink Genomic DNA Mini Kit (Thermo Fisher, K82001) following the manufacturer’s instructions. gDNA PCR to amplify LwCas13 was performed using Q5 high-fidelity DNA polymerase (NEB) and the primer pair LwaCas13a-145-SeqF cagcctgaagtacagcttcga and LwCas13-320-R tatcgttgctgatgttgctca for the 5’-end and LwaCas13a-1035-Seq-For ctgaagcaggaaaaaaaggac. 3-H8 Puro_start-seqR caccgtgggcttgtactcg for the 3’-end. PCR products (5’-end 530 bp and 3’-end 735 bp were visualised by capillary electrophoresis QIAxcel (Qiagen).

### Lentiviral packaging and transduction

The lentiviral plasmids, LV-Cas13a-T2A-Puro, LV-Cas13a-Puro-TetR and Tet-LV- *KRAS*-gRNA-13a-GFP, were packaged into lentivirus by VectorBuilder. For LV-LwaCas13a-Puro, HaCaT and HEK293T cells were transduced to a 2.45 and 3 MOI respectively. For *KRAS*-gRNA cells were transduced to a 17 MOI.

### Immuno blot and microscopy

LwaCas13a expression was analyzed by immunoblot using mouse anti-HA primary antibody and IRDye infrared dye (Li-Cor Biosciences)-conjugated anti-mouse secondary antibody. Proteins were visualized using a LI-COR Odyssey CLx scanner and analyzed using ImageStudio v 5.2.5. mCherry and GFP were visualized using a ZOE fluorescent cell imager (Bio-Rad).

### RNA isolation, RT-qPCR and RNA-seq

RNA isolation was performed using an HP RNA isolation kit (Roche E3101K) following the manufacturer’s instructions. Quantitative RT-PCR was performed using 30 ng of total RNA and One-Step RT-PCR kit (Qiagen, 210212). Data were analyzed using the STEPOnePlus 1 Real-Time PCR system (Thermo Fisher Scientific). Paired t-test was performed using Prism 10 software. The following primer pairs were used for RT-qPCR.

*KRAS*, ADS6340 gaggcctgctgaaaatgactg and ADS6341 attactacttgcttcctgtagg. *Neomycin*, Neo-RT-F3 (cctgtcatctcaccttgctc), Neo-RT-R2 (gctcttcgtccagatcatcc), *MALAT1*, MALAT1-RT-F (aaagcccaaatctcaagcgg), MALAT-RT-R (ttcaacccaccaaagacctc), and house keeping *ACTB*, ACTB-qPCR-F (aagatcaagatcattgctcctcc), ACTB-qPCR-R (ggactcgtcatactcctgct), *TOPO*, TOP-qPCR-F (cgaatcaagggtgagaagga), and TOP1-qPCR-R (cggactttcacttctttggac) and *GAPDH*, ADS2184/(acagtcagccgcatcttctt) and ADS2185 (ttgattttggagggatctcg).

### RNA-seq

Unmapped paired-end sequences from an Illumina HiSeq4000/NovaSeq 6000 sequencer were tested using FastQC ( http://www.bioinformatics.babraham.ac.uk/projects/fastqc/). Sequence adapters were removed, and reads were quality-trimmed using Trimmomatic_0.39 (PMID: 24695404). The reads were mapped against the reference human genome (hg38), and counts per gene were calculated using annotations from GENCODE 43 ( http://www.gencodegenes.org/) using STAR_2.7.7a (PMID: 23104886). Normalization, Principal Components Analysis, and differential expression were calculated using DESeq2_1.40.2 (PMID:25516281). Adjusted *p*-values were corrected for multiple testing (Benjamini and Hochberg method). Heatmaps were drawn with complexHeatmap v2.16.0 (PMID: 27207943). Gene ontology enrichment was performed using clusterProfiler v4.8.3 (PMID: 34557778) and Enrichr v3.2 (PMID: 27141961). The RNA-seq data have been submitted to ArrayExpress (accession no. E-MTAB-14334). Analised data was submitted to Biostudies (accession no. S-BSST2016)

### Differential gene expression analysis

Differential gene expression analysis was performed using the DESeq2 package in R, on a dataset containing 62,703 transcripts. This dataset was filtered down to 20,044 protein-coding transcripts. Differentially expressed mRNAs were identified if they had a log fold-change value of less than -1 when comparing the *MALAT1* vs. untreated groups and the *Neo* vs. untreated groups. To ensure the robustness of our dataset, mRNAs with zero read counts were excluded. The dataset was further refined by focusing on the top 200 mRNAs with the highest absolute fold change values. These filtering steps narrowed the list of candidate genes with the greatest potential to provide meaningful signals.

### Motif Identification with MEME

To identify sequence motifs associated with the downregulated transcripts, *de novo* motif discovery was performed using MEME (v5.5.8) ( Bailey & Elkan, 1994) and HOMER (v5.1). The analysis was based on the top 200 mRNAs showing the greatest absolute log fold change values (LFC < –1) and adjusted p-values < 0.05 in both the MALAT1 and Neo groups (as described in the Differential Gene Expression Analysis section). MEME and HOMER were used to search for motifs enriched in this downregulated set compared to a background group of unaffected control sequences, generated from 200 mRNAs with the lowest absolute LFC values and highest adjusted p-values. Corresponding cDNA sequences for each mRNA were extracted using the Ensembl BioMart tool (GRCh38 release). For MEME, motif discovery was performed using the ZOOPS model (zero or one occurrence per sequence), with a motif width range of 7–12 bp and all other parameters left at default settings. HOMER was also run with default de novo motif discovery parameters using the same sets of downregulated and unaffected background sequences. Motifs were ranked by statistical significance (E-value < 0.05 for MEME; p < 0.05 for HOMER).

## Results

### Stable expression of LwaCas13a is compromised in HaCaT and HEK293T cells

**Figure 1.**
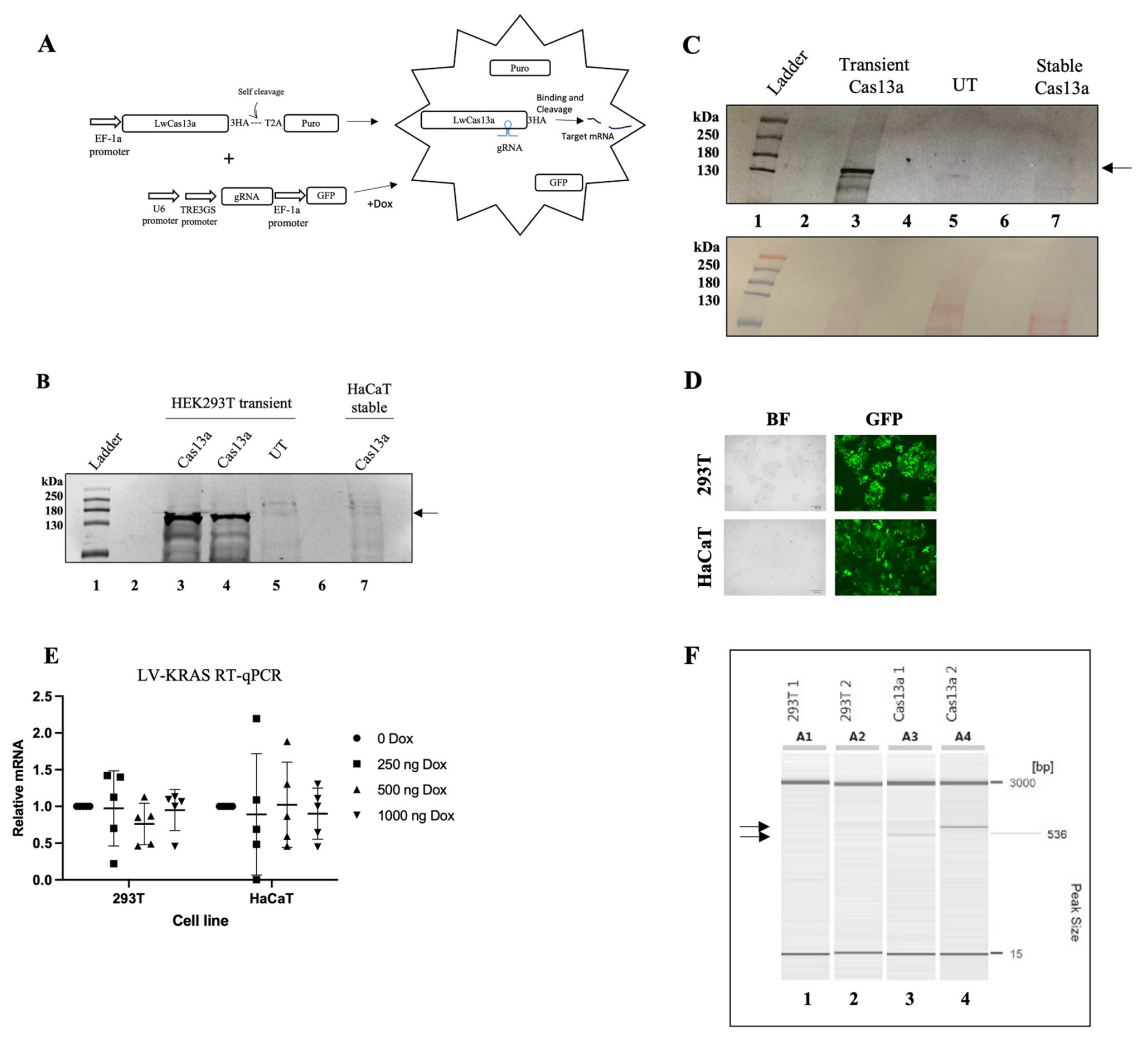
LwaCas13a expression is silenced in HaCaT and HEK293T cells. ( **A**) Diagram of the strategy to down-regulate gene expression using LwaCas13a. Cells constitutively expressing LwaCas13a. Dox-inducible gRNA expression. Puro-puromycin, 3HA- three hemagglutinin tags, 2TA- self-cleaving peptide from *Thosea asigna* virus, gRNA- guide-RNA, GFP- green fluorescent protein. ( **B**) Immunoblot detecting LwaCas13a using an anti-HA primary antibody. *Lane 1*- protein ladder, *2*- empty, *3 and 4-* LwaCas13a transiently-transfected in HEK293T cells, *5-* un-transfected HEK293T, *6-* empty, *7*- LwaCas13a transduced and selected HaCaT. Arrow indicates LwaCas13a position on the blot. ( **C**) LwaCas13a was detected using an anti-HA primary antibody. *Lane 1-* protein ladder, *2-* empty, *3-* transiently expressed LwaCas13a, *4-* empty, *5-* un-transfected HEK293T cells, *6-* empty, *7-* passage five, stable LwaCas13a HEK293T cells. Arrow indicates LwaCas13a position on the blot. Bottom, Ponceau S stain of the nitrocellulose membrane used for the immunoblot. ( **D**) HEK293T (top) or HaCaT (bottom) cells after 48 h of transduction (+Dox) with the Dox inducible gRNA targeting *KRAS*. ( *E*) RT-qPCR analysis of the expression of *KRAS* in LwaCas13a-HEK293T or LwaCas13a-HaCaT cells transduced with Dox-inducible *KRAS* gRNA. Data of five independent experiments using different Dox concentration is presented. ( *F*) Capillary electrophoresis of LwaCas13a gDNA-PCR. *Lane 1-*un-transduced HEK293T 5’-LwaCas13a *2-* un-transduced HEK293T 3’-LwaCas13a *3-* transduced HEK293T 5’-LwaCas13a *4-* transduced HEK293T 3’-LwaCas13a. Arrows indicate the sizes of the PCR products corresponding to LwaCas13a.

We first attempted to establish a human keratinocyte HaCaT cell line that constitutively expresses LwaCas13a using lentivirus. The integrated transgene would simultaneously express LwaCas13a and puromycin under the control of the common E1Fα promoter by the presence of the self-cleaving peptide from *Thosea asigna* virus (T2A) downstream of LwaCas13a. The T2A peptide would allow the expression of equal amounts of the linked proteins, and because of its small size, it would not interfere with protein function ( Wang *et al.*, 2015) ( Figure 1A). Transduced HaCaT cells were selected with puromycin. However, LwaCas13a expression was not detected by immunoblot against the HA tag of LwaCas13a ( Figure 1B, lane 7). To determine whether this was due to incompatibility of expression in the HaCaT cell line, we transduced and puromycin selected an alternative line, HEK293T cells, with the same lentivirus. LwaCas13a expression in HEK293T cells was confirmed by immunoblot ( Figure 1C, lane 3). However, after approximately five passages, we could not detect LwaCas13a in the puromycin-resistant cells ( Figure 1C, lane 7). To test whether LwaCas13a was still present in the cells but at levels that could not be detected by immunoblot, we tested its activity. For this, we transduced a doxycycline (Dox) inducible gRNA previously reported to knock down *KRAS* ( Abudayyeh *et al.*, 2017). GFP, coded on the same construct but expressed from the EF1α promoter, was used to monitor the efficiency of transduction ( Figure 1D). Cells were transduced with high efficiency and cell toxicity was not observed ( Figure 1D). Transduced cells were treated with increasing concentrations of Dox for 48 h. Knock-down of *KRAS* was analysed by Reverse Transcription quantitative Polymerase Chanin Reaction (RT-qPCR). However, no significant difference on *KRAS* mRNA levels (paired t-test) was observed between the Dox treated and un-treated cells. Suggesting LwaCas13a was not active in the puromycin resistant cells ( Figure 1E). Integration of the LwaCas13a trans-gene into the cell genome was confirmed by polymerase chain reaction (PCR). The PCR products were only observed from the genomic DNA of the LwaCas13a transduced cells ( Figure 1F). These results suggest that the cells silenced LwaCas13a because it was either toxic or because incomplete cleavage of the T2A peptide interfered with the stability of LwaCas13a. Negative effects of the T2A on protein stability has been previously reported for other proteins like KLF4 ( Chng *et al.*, 2015; Reinhardt *et al.*, 2020). 

### Targeting of mammalian RNAs by LwaCas13a results in cell toxicity and death

**Figure 2.**
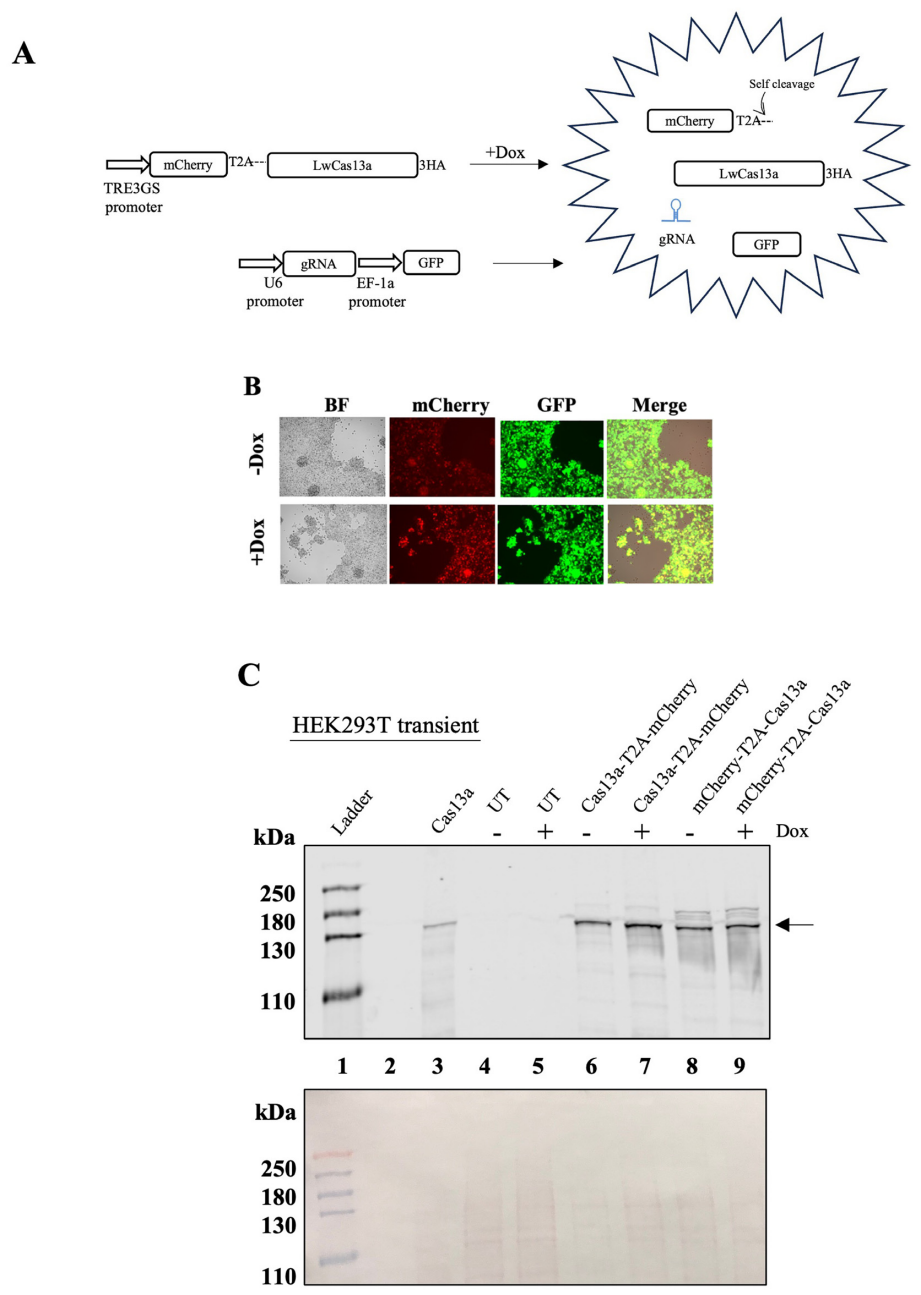
Inducible expression of LwaCas13a in HEK293T cells. ( **A**) Diagram of the system used to express LwaCas13a in HEK293T. After treatment with Dox, cell express mcherry-LwaCas13a. gRNA and GFP are constitutively expressed. Puro-puromycin, 3HA- three hemagglutinin tags, 2TA- self-cleaving peptide from *Thosea asigna* virus, gRNA- guide-RNA, GFP- green fluorescent protein, Dox- doxycycline. ( **B**) HEK293T cells after 48 h of transfection with mCherry-LwaCas13a and gRNA targeting Neomycin without (top) or with doxycycline (bottom). ( **C**) Top, LwaCas13a was detected using an anti-HA primary antibody. *Lane 1-* protein ladder *2-*empty *3-* LwaCas13a *4-* un-transfected HEK293T cells *5-* un-transfected HEK293T cells +Dox *6-* inducible LwaCas13a-T2A-mCherry *7-* inducible LwaCas13a-T2A-mCherry +Dox *8-* inducible mCherry-T2A-LwaCas13a *9-* inducible mCherry-T2A-LwaCas13a +Dox. Arrow indicates LwaCas13a position on the blot. Bottom, Ponceau S stain of the nitrocellulose membrane used for the immunoblot.

To overcome any interference from the T2A peptide or from the constitutive expression of LwaCas13a, we designed and cloned a LwaCas13a-doxycycline (Dox)-inducible expression system (Tet-ON) and added the T2A sequence either upstream or downstream of LwaCas13a. To identify the LwaCas13a expressing cells, mCherry was linked to the amino- or carboxyl-terminal end of LwaCas13a by the T2A peptide ( Figure 2A). Despite growing the cells in Tet-free serum, basal expression of mCherry and LwaCas13a was observed in the absence of Dox ( Figure 2B top panel and Figure 2C lanes 6 and 8). Such leaky expression of the TetON system has been previously reported ( Costello *et al.*, 2019). The addition of Dox increased the expression of both proteins ( Figure 2C, lanes 7 and 9 and Figure 2B bottom panel). Expression of LwaCas13 was observed in both constructs independently of the location, amino- or carboxyl- end, of the T2A peptide ( Figure 2C, lanes 7 and 9). To test the activity of LwaCas13a in our system, we targeted *Neomycin* (Neo), which is expressed by HEK293T cells but is not essential for the survival of these cells. As an additional control, we also targeted lncRNA *MALAT1,* previously targeted in HEK293T ( Abudayyeh *et al.*, 2017). For this, we co-transfected the Dox-inducible-LwaCas13a and a plasmid encoding the gRNA under the control of the U6 promoter ( Figure 2A and 2B). This plasmid also expressed the eGFP protein under control of the EF-1α promoter. Surprisingly, the expression of LwaCas13a, even at low, but detectable by immunoblot levels (-Dox), and in presence of gRNAs, caused significant cell toxicity, with cell death in co-transfected cells within 48 h post-transfection.

### High trans-collateral activity of LwaCas13a in human cells

**Figure 3.**
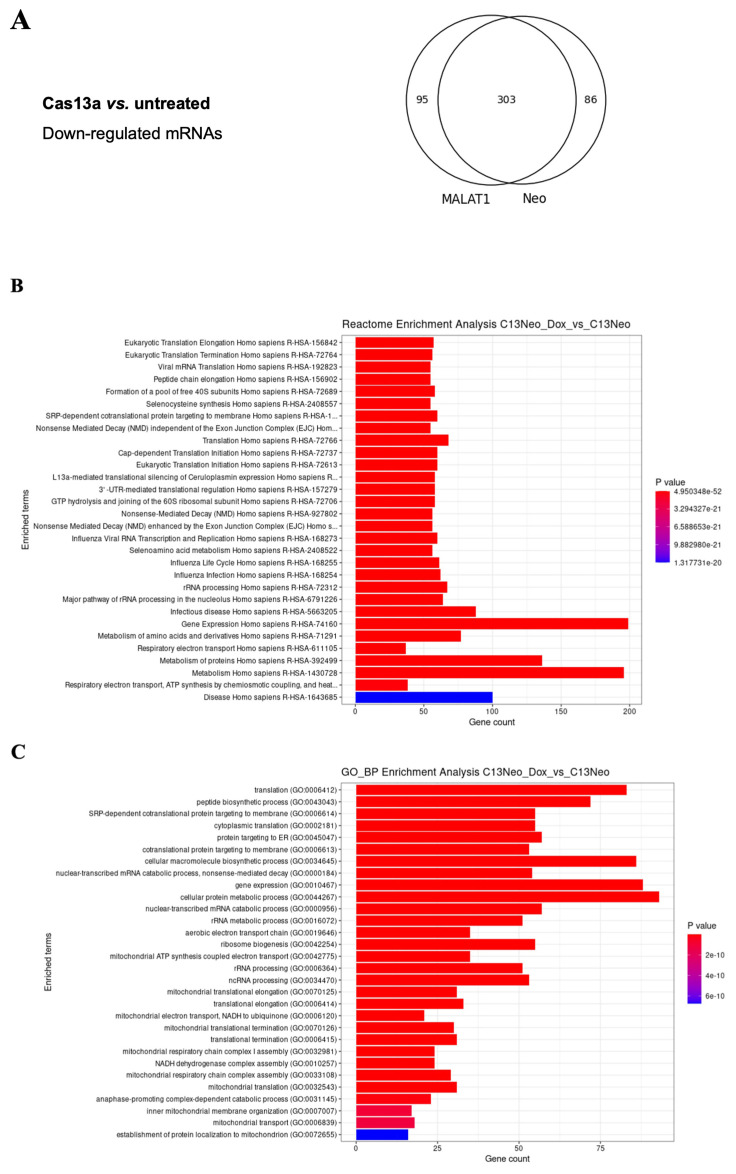
The trans-collateral activity of LwaCas13a un-specifically targets cellular transcripts. ( **A**) Venn diagram showing the overlap of downregulated mRNAs between MALAT1 and Neo gRNAs transfected cells. ( **B**) Reactome enrichment analysis of cellular functions affected by LwaCas13a, Neo-gRNA -/+ Dox. P value represents the statistical significance of a pathway being enriched with genes down-regulated by LwaCas13a. ( **C**) Gene ontology (GO) enrichment analysis of biological pathways (BP) affected by LwaCas13a, Neo gRNA -/+ Dox. P value represents the statistical significance of a pathway being enriched with genes down-regulated by LwaCas13a.

**Figure 4.**
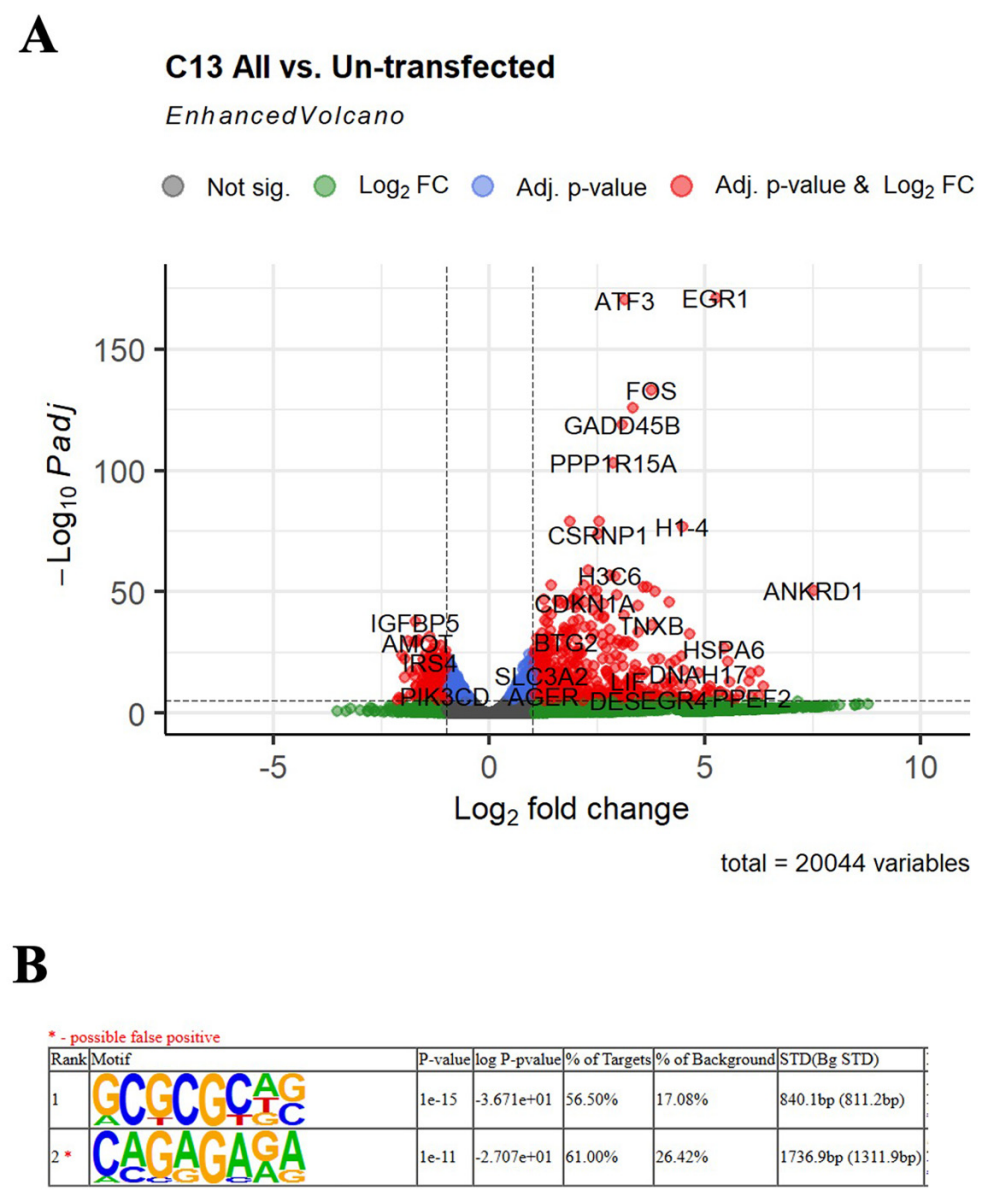
LwaCas13a downregulated genes. ( **A**) Volcano plot of the transcripts that were up- or down-regulated in cells transfected with LwaCas13a. X axis shows the Log2 fold change in expression on LwaCas13a treated samples compared to the un-treated sample. Y axis shows the -Log10 adjusted p-value. FC- fold change, P- p-value. ( **B**) HOMER motif discovery of the top 200 down-regulated mRNAs in the LwaCas13a samples compared to the un-treated sample. * Denotes possible false positive motif. STD(bg STD) means Standard deviation of motif occurrence (bp) away from the center of the sequence for background set.

In bacteria, once LwaCas13a has found and cleaved its specific target, which is complementary to the gRNA, the trans-collateral activity of LwaCas13a cleaves any single-stranded RNA independently of its complementarity to the gRNA. Initial reports of Cas13 in eukaryotic cells claimed that the trans-collateral activity was absent in this system ( Abudayyeh *et al.*, 2017). However, several groups have recently observed the toxic effects of Cas13a ( Ai *et al.*, 2022; Shi *et al.*, 2021; Wang *et al.*, 2019). Therefore, we investigated whether the observed cytotoxic effect was due to trans-collateral activity of LwaCas13a. For this, we performed RNAseq on cells co-transfected with mCherry-T2A-Cas13a and gRNA targeting *Neo* or *MALAT1*. As a consequence of the stress the cells undergo when co-transfected with LwaCas13a and the gRNAs, high quality total RNA of only one experiment was isolated. To analyse the data, due to the lack of biological repeats, we merged and compared all the gRNA treated samples against the untransfected sample and chose the ones with log2 fold change(LFC) below -1 (for downregulation) and an adjusted p-value below 0.05. Of the 20,044 unique coding RNAs sequenced, 95 and 86 were downregulated in gRNA-Neo or gRNA-MALAT1, respectively, and 303 downregulated mRNAs were common to both gRNA-Neo and gRNA-MALAT1 samples compared to the untransfected sample ( Figure 3A and Figure 4A, EAM-extended data-table 1 and 2, Biostudies accession no. S-BSST2016). To verify that the downregulated mRNAs were not targeted by complementary gRNAs, we used a custom BLAST search and pairwise alignmet scripts to align the gRNA sequences with cDNA from the downregulated mRNAs but did not find complementary matches with significant query coverage or alignment scores. These results strongly suggest that the trans-collateral activity of LwaCas13a is responsible for the downregulation of mRNAs.

We then decided to investigate whether LwaCas13a cleaves eukaryotic mRNAs independently of sequence or if it prefers uridine-rich motifs as it does *in vitro* ( Abudayyeh *et al.*, 2017; Abudayyeh *et al.*, 2016; Gootenberg *et al.*, 2018). To answer this, we performed a *de novo* discriminated motif analysis on the top 200 downregulated mRNAs for the LwaCas13a samples, comparing them to the motifs found in the un-affected mRNAs. A GC-rich sequence was found in more than 56% of the downregulated mRNAs, suggesting LwCas13a preferentially cuts RNAs with this motif ( Figure 4B). However, many mRNAs lacking such motif were targeted by LwaCas13a. Taken together, these results suggest that similar to its activity in bacteria and *Archaea*, activated LwaCas13a indiscriminately cleaves RNA present in the cell independently of their sequence or complementarity to the gRNA. This indiscriminate activity affects the expression of mRNAs required for normal cell function and survival. Examples of these essential mRNAs cleaved by LwaCas13a include *SOX3*, *DDIT4*, *MYC* and *CCNI2*. Many non-specifically targeted genes had critical functions in RNA processing, protein synthesis, and metabolic pathways, likely contributing to the observed cell death ( Figure 3B, 3C). In addition, genes involved in cellular stress response, such as *FOS*, *ATF3*, *GADD45B* were upregulated in cells expressing LwaCas13a-gRNA ( Figure 4A and Table 1), confirming the activation of the stress response in the presence of LwaCas13a-gRNA.

**Table 1.** Top five upregulated genes in cells expressing LwaCas13a.

Gene symbol	Function	Log2 FC	P value
EGR1	Early growth response transcription factor, regulates cell growth and DNA-damage response	5.25	2.24E-175
FOS	Transcription factor activating cell proliferation as a part of AP-1 complex downstream of mitogen-activated kinase pathway (MAPK)	3.76	8.31E-138
ATF3	Activating transcription factor 3, upregulated in response to cellular stress	3.13	4.6E-175
GADD45B	Growth arrest and DNA-damage-inducible (isoform B).	3.08	2.16E-123
PPP1R15A	Protein phosphatase P1 regulatory subunit 15A activates eucaryotic initiation factor 2 alpha and protein translation	2.86	1.73E-107

* Log2FC is the log2 fold-change in expression compared to the un-treated cells $ p-adj value was calculated, comparing all the samples treated with LwaCas13a against the un-treated sample

## Conclusion

In our hands, LwaCas13a demonstrated high non-specific activity and led to cell toxicity when used as a transcript-targeting system. Our data indicates that LwaCas13a is poorly suited for RNA interference applications. These findings are also supported by reports on the off-target effects of LwaCas13a in glioma cells ( Wang *et al.*, 2019). However, some groups have reported a high specificity of knockdown by LwaCas13a ( Abudayyeh *et al.*, 2017). This controversy does not seem to be exclusive for LwaCas13a. Other groups have also shown high levels of off-targets of RxCas13d in *Drosophila*, HeLa, and HEK293T cells ( Ai *et al.*, 2022; Shi *et al.*, 2021), whereas others have shown near no off-targets of RxCas13d in HEK293FT, U2OS, and iPSC lines ( Konermann *et al.*, 2018). This may indicate collateral activity in cells to be a cell, target type, and Cas13- specific effect.

Due to the presence of the trans-collateral activity of LwaCas13a in mammalian cells, we do not recommend its use to specifically downregulate RNAs. However, the transcollateral activity of LwaCas13 can be exploited for *in vitro* diagnosis ( Kellner *et al.*, 2019). Alternatively, the catalytically inactive form of Cas13 (dCas13) can be used to track specific RNAs in live cells ( Yang *et al.*, 2019).

## Ethics and consent

Ethical approval and consent were not required.

## Data Availability

The RNA-seq data have been submitted to ArrayExpress (accession no. E-MTAB-14334). https://www.ebi.ac.uk/biostudies/arrayexpress/studies/E-MTAB-14334?query=E-MTAB-14334 ( Zeef (2025).) Tables I and II have been submitted to Biostudies (accession no. S-BSST2016) https://www.ebi.ac.uk/biostudies/studies/S-BSST2016 ( Aguilar-Martínez (2025))
