# Supplementary material for: LwaCas13a indiscriminately targets the human transcriptome
Source: Wellcome Open Res. 2026 Apr 17;10:242. Originally published 2025 May 16. [Version 3] doi: 10.12688/wellcomeopenres.23656.3 (PMC13133622; doi:10.12688/wellcomeopenres.23656.3)
Supplement: Peer review history

# Reviewer report: Reviewer response for version 3

**DOI:** 10.21956/wellcomeopenres.28842.r153524

**Published:** 2026-04-30

**Authors:** Erin Doherty

**Affiliations:** 1 University of California, Berkeley, california, USA

The changes to the text and new experiments appropriately address my initial set of comments.

Is the work clearly and accurately presented and does it cite the current literature?

Partly

If applicable, is the statistical analysis and its interpretation appropriate?

Yes

Are all the source data underlying the results available to ensure full reproducibility?

Yes

Is the study design appropriate and is the work technically sound?

Partly

Are the conclusions drawn adequately supported by the results?

No

Are sufficient details of methods and analysis provided to allow replication by others?

Yes

Reviewer Expertise:

Prokaryotic immunity, CRISPR tool development, RNA editing

I confirm that I have read this submission and believe that I have an appropriate level of expertise to confirm that it is of an acceptable scientific standard.

# Reviewer report: Reviewer response for version 2

**DOI:** 10.21956/wellcomeopenres.27736.r141175

**Published:** 2026-01-23

**Authors:** Diego di Bernardo, Virginia Fusco

**Affiliations:** 1 University of Naples Federico II, Naples, Italy

The authors are addressing the potential toxicity and off-targeting effects associated with the expression of Cas13 in a mammalian cell line. This report builds on previous studies that have examined the off-target activity  in bacteria as well as in certain mammalian systems. However, several aspects require further work. In particular, the conclusion drawn from the RNA-sequencing data is not very strong, and the discussion should acknowledge the limitations of this analysis or, alternatively, include additional experiments to increase the number of cases for which RNA-seq can be performed.

 In more details:

- Figure 1C: Regarding the results in panel C, it is unclear whether the CAS13 expression in lane 3 is transient or stable. From the text: *“To determine whether this was due to incompatibility of expression in the HaCaT cell line, we transduced and puromycin-selected an alternative line, HEK293T cells, with the same lentivirus. LwaCas13a expression in HEK293T cells was confirmed by immunoblot (Figure 1C, lane 3)”*, it appears that transduced and selected cells were tested, but in the figure and caption, CAS13 is marked as “Transient.” I think the manuscript would benefit from a clearer comparison between: transient transfection using an amount of material simulating the MOI used for the transduction, and the transient immunoblot compared to that from stable LwaCas13a-expressing lines, both at the first and fifth passage.
  If these data are already present, they should be presented more clearly.
- Figures 1E and 2A: For both experiments, I suggest including a comparison with wild-type cells. The reported Pol II promoter activity of the LTR could also influence U6 promoter activity, potentially causing excessive leakiness. A similar consideration applies to the doxycycline-inducible Cas experiments later in the manuscript.
- RNA-seq and collateral effects: Number of samples has to be increased or at least limitations should be discussed in the discussion. Also  reducing the treatment time may help to consistently isolate high-quality RNA and better confirm the findings. Additionally, I think a more thorough analysis of the samples should be conducted to ensure that the observed effects are indeed due to off-target activity of CAS13.

Minor:

1. In the introduction: change Clustered regularly interspaced short palindromic repeat  to Clustered Regularly Interspaced Short Palindromic Repeats (CRISPR)
2. Change Polymerase Chanin Reaction (RT-qPCR) to Polymerase Chain Reaction (RT-qPCR)
3. change pairwise alignmet scripts to  pairwise alignment scripts
4. Twice original to original
5. In cell culture methods: change CO ^2  ^to CO _2_

Is the work clearly and accurately presented and does it cite the current literature?

Partly

If applicable, is the statistical analysis and its interpretation appropriate?

Yes

Are all the source data underlying the results available to ensure full reproducibility?

Yes

Is the study design appropriate and is the work technically sound?

Partly

Are the conclusions drawn adequately supported by the results?

Partly

Are sufficient details of methods and analysis provided to allow replication by others?

Partly

Reviewer Expertise:

synthetic biology, computational biology.

We confirm that we have read this submission and believe that we have an appropriate level of expertise to confirm that it is of an acceptable scientific standard, however we have significant reservations, as outlined above.

# Response

**Published:** 2026-03-04

**Authors:** Elisa Aguilar-Martinez

**Affiliations:** University of Manchester, UK

The authors are addressing the potential toxicity and off-targeting effects associated with the expression of Cas13 in a mammalian cell line. This report builds on previous studies that have examined the off-target activity in bacteria as well as in certain mammalian systems. However, several aspects require further work. In particular, the conclusion drawn from the RNA-sequencing data is not very strong, and the discussion should acknowledge the limitations of this analysis or, alternatively, include additional experiments to increase the number of cases for which RNA-seq can be performed.

 In more details:

- Figure 1C: Regarding the results in panel C, it is unclear whether the CAS13 expression in lane 3 is transient or stable. From the text: *“To determine whether this was due to incompatibility of expression in the HaCaT cell line, we transduced and puromycin-selected an alternative line, HEK293T cells, with the same lentivirus. LwaCas13a expression in HEK293T cells was confirmed by immunoblot (Figure 1C, lane 3)”*, it appears that transduced and selected cells were tested, but in the figure and caption, CAS13 is marked as “Transient.”
- **We have modified the text to make it clear that LwaCas13a detected in the immuno blot in Figure 1C, lane 3 is after transient transfection and lane 7 is the transduced and selected line.**

 

- I think the manuscript would benefit from a clearer comparison between: transient transfection using an amount of material simulating the MOI used for the transduction, and the transient immunoblot compared to that from stable LwaCas13a-expressing lines, both at the first and fifth passage. If these data are already present, they should be presented more clearly.
- It is an interesting suggestion. However, the transient transfection was caried out using the plasmid packed into lentivirus and used for the transductions. This shows that the construct is working. In addition, we obtained cells resistant to Puromycin, in both HaCaT and HEK293T cells, showing a successful transduction.
- Figures 1E and 2A: For both experiments, I suggest including a comparison with wild-type cells. The reported Pol II promoter activity of the LTR could also influence U6 promoter activity, potentially causing excessive leakiness. A similar consideration applies to the doxycycline-inducible Cas experiments later in the manuscript.
- **As pointed by the referee the dox system can leak even in tet-free FBs as we show in figure 2C. However, we only included the sample without Dox and not wild-type cells, because the RT-qPCR CT values observed for *KRAS* 0 Dox, were consistent with previous experiments performed in the same HEK293T, where cells transfected with gRNAs targeting luciferase have a *KRAS* CT value of 21-23 and consistent values for the house keeping genes as well. **

 

- RNA-seq and collateral effects: Number of samples has to be increased or at least limitations should be discussed in the discussion. Also  reducing the treatment time may help to consistently isolate high-quality RNA and better confirm the findings.
- **As included in the results and discussion section of the manuscript, we tried to obtain more samples to have biological replicates of the experiment. However, the quality of the RNA was low and could not be submitted for RNAseq. Reducing treatment time might increase the number of viable cells. However, we started to observe cell dead 24 h post transfection. Also, shorter time might not achieve KD of the targets. Therefore, using LwaCas13a for KD will not be appropriate. This is in accordance with an increasing number of reports showing transactivation activity of Cas13 in mammalian cells. We have referred to these studies in the manuscript.**

 

- Additionally, I think a more thorough analysis of the samples should be conducted to ensure that the observed effects are indeed due to off-target activity of CAS13.
- **We thank the referee for suggesting a more thorough analysis and agree this would be an interesting development. However, due to the limited scope of the project, we focused in the results of our thorough analysis of gRNAs and Cas13a activity in the presented system. We examined potential complementary matches between the downregulated transcripts and the gRNAs and also assessed the presence of uridine-rich and *de novo* motifs within the downregulated transcrips; however, no such features were identified. These findings suggest that the transactivator activity of LwaCas13a, previously reporte in bacteria and Archae, may also be present in mammalian cells.**

**Minor: all minor changes have been incorporated to the manuscript.**

# Reviewer report: Reviewer response for version 2

**DOI:** 10.21956/wellcomeopenres.27736.r140457

**Published:** 2026-01-02

**Authors:** James William Bryson

**Affiliations:** 1 University of Copenhagen, Copenhagen, Denmark

The revision is improved in clarity and I appreciate several concrete changes, including the addition of ponceau staining for the Western blots, expanded methodological detail and addition of the KRAS gRNA experiment intended to assess whether the stable/selected lines retain functional Cas13a activity.

 That said, I think two key issues remain that materially affect what can be concluded from the current data, and these should be addressed through scaling back the claims made and/or additional controls.

 For the RNA-seq, the conclusions and statistical language should better match what the design supports. The revised text indicates that high-quality total RNA was obtained from only a single experiment and that analyses were adapted accordingly by “merging” samples and comparing to an untransfected control. In this setting, inferential statements supported by adjusted p-values (and any downstream enrichment statistics that depend on those calls) are difficult to justify, because dispersion/variance cannot be robustly estimated without biological replication.

 I think the RNA-seq can still be valuable as descriptive evidence consistent with broad transcriptomic disruption under the experimental condition, but the manuscript should explicitly frame these results as exploratory/descriptive rather than definitive evidence of trans-collateral cleavage. 

 Here I recommend clearly stating sample numbers and what constitutes a biological replicate for each condition, and revising language throughout Results/Conclusion that currently implies statistical robustness (e.g. “significantly downregulated”, “strongly suggest”, “responsible for”) to wording consistent with an n=1 (e.g. “we observed”, “consistent with”, etc), unless additional replicates are provided.

 Regarding the attribution of toxicity/collateral activity the claims seem too strong given the controls/data available. The revision better documents that toxicity is observed after co-transfection of Cas13a + guide constructs. However, as written, the manuscript still leans toward causal attribution to collateral activity rather than presenting it as a plausible mechanism among alternatives (including stress-driven transcriptome shifts in dying cells). To support the stronger mechanistic claim, I would still expect at least one clear control that separates “Cas13a activation/catalysis” from “expression/transfection burden”, for example either Cas13a + non-targeting guide (or scaffold-only) or catalytic-dead Cas13a + same guides.

 If such experiments are not feasible, then the manuscript should temper the mechanistic conclusion and explicitly acknowledge that stress response and/or general toxicity could contribute substantially to the observed transcript changes.

 Summary recommendation

 The paper is substantially improved, but I recommend revising the strength of claims to match the current evidence base (particularly the RNA-seq), correcting any inconsistent reporting of RNA-seq results, and either adding a minimal control to support causal attribution of toxicity/collateral activity or reframing the conclusion as observational and context-specific (“in our hands/under these conditions”) rather than general (“unsuitable tool”).

Is the work clearly and accurately presented and does it cite the current literature?

Partly

If applicable, is the statistical analysis and its interpretation appropriate?

Partly

Are all the source data underlying the results available to ensure full reproducibility?

Yes

Is the study design appropriate and is the work technically sound?

No

Are the conclusions drawn adequately supported by the results?

No

Are sufficient details of methods and analysis provided to allow replication by others?

Partly

Reviewer Expertise:

CRISPR tools, sequencing method development, bioinformatics

I confirm that I have read this submission and believe that I have an appropriate level of expertise to confirm that it is of an acceptable scientific standard, however I have significant reservations, as outlined above.

# Response

**Published:** 2026-03-04

**Authors:** Elisa Aguilar-Martinez

**Affiliations:** University of Manchester, UK

The revision is improved in clarity and I appreciate several concrete changes, including the addition of ponceau staining for the Western blots, expanded methodological detail and addition of the KRAS gRNA experiment intended to assess whether the stable/selected lines retain functional Cas13a activity.

 That said, I think two key issues remain that materially affect what can be concluded from the current data, and these should be addressed through scaling back the claims made and/or additional controls.

 For the RNA-seq, the conclusions and statistical language should better match what the design supports. The revised text indicates that high-quality total RNA was obtained from only a single experiment and that analyses were adapted accordingly by “merging” samples and comparing to an untransfected control. In this setting, inferential statements supported by adjusted p-values (and any downstream enrichment statistics that depend on those calls) are difficult to justify, because dispersion/variance cannot be robustly estimated without biological replication.

 I think the RNA-seq can still be valuable as descriptive evidence consistent with broad transcriptomic disruption under the experimental condition, but the manuscript should explicitly frame these results as exploratory/descriptive rather than definitive evidence of trans-collateral cleavage. 

 Here I recommend clearly stating sample numbers and what constitutes a biological replicate for each condition, and revising language throughout Results/Conclusion that currently implies statistical robustness (e.g. “significantly downregulated”, “strongly suggest”, “responsible for”) to wording consistent with an n=1 (e.g. “we observed”, “consistent with”, etc), unless additional replicates are provided.

 Regarding the attribution of toxicity/collateral activity the claims seem too strong given the controls/data available. The revision better documents that toxicity is observed after co-transfection of Cas13a + guide constructs. However, as written, the manuscript still leans toward causal attribution to collateral activity rather than presenting it as a plausible mechanism among alternatives (including stress-driven transcriptome shifts in dying cells). To support the stronger mechanistic claim, I would still expect at least one clear control that separates “Cas13a activation/catalysis” from “expression/transfection burden”, for example either Cas13a + non-targeting guide (or scaffold-only) or catalytic-dead Cas13a + same guides. Cells do not undergo stress when transiently transfected with LwaCas13a in absence of gRNA (Figure 1B, 1C and 2C) or in cells with gRNA but with un-detectable expression of LwCas13a (Figure 1E). Cell stress and dead was only seen, in our hands, when cells were co-transfected with LwaCas13a and gRNA.

 If such experiments are not feasible, then the manuscript should temper the mechanistic conclusion and explicitly acknowledge that stress response and/or general toxicity could contribute substantially to the observed transcript changes.

**We have modified the text to include that stress response and general toxicity could contribute to the changes in transcription.**

 Summary recommendation

 The paper is substantially improved, but I recommend revising the strength of claims to match the current evidence base (particularly the RNA-seq), correcting any inconsistent reporting of RNA-seq results, and either adding a minimal control to support causal attribution of toxicity/collateral activity or reframing the conclusion as observational and context-specific (“in our hands/under these conditions”) rather than general (“unsuitable tool”).  

**We thank the referee for the comments. With this manuscript we are sharing our experience while using LwaCas13a in mammalian cells. Reporting “negative” results is challenging, especially when cell death is observed while performing the experiments. We have analysed and reported our findings in the best possible way. Across the manuscript we have already stated why there are no biological replicates for the RNAseq experiment. Also, we have written, “the results suggest”, “In our hands”, “our data indicates” “we do not recommend” (see conclusions). We have considered the referee’s comments and removed words like “strongly” and although, we cite works from other groups that have also observed off-target effects of LwaCas13a, we included more times the phrase “in our hands” in the manuscript.**

# Reviewer report: Reviewer response for version 2

**DOI:** 10.21956/wellcomeopenres.27736.r141171

**Published:** 2025-12-31

**Authors:** Erin Doherty

**Affiliations:** 1 University of California, Berkeley, california, USA

This work illustrates technical challenges in using and applying LwaCas13a which would be relevant to researchers using this system and potentially other orthologs. However, as stated, the paper lacks the necessary controls to support the conclusions as they are written.

 In Figure 1, the authors suggest that “the cells silenced LwaCas13a because it was either toxic or because incomplete cleavage of the T2A peptide interfered with stability of LwaCas13a.” The concern with the T2A peptide influencing stability is understood, and experiments in Figure 2 convincingly rule this out as the reason for lack of detectable Cas13a expression in Figure 1. Lack of KRAS knockdown supports the lack of robust Cas13a activity under these conditions but does not rule out the possibility that LwaCas13a is simply lowly expressed in these conditions. Given that cells are puromycin resistant and Puro is downstream of Cas13, it is possible that expression is sustained at a level high enough to confer puromycin resistance but not robust Cas13 knockdown. This finding still retains its usefulness to the field—that integration in this context is not conducive to robust knockdown. However, I think that the authors cannot draw a definitive conclusion on this mechanism based on the existing data.

 The authors report LwaCas13a-induced toxicity, consistent with their report of high collateral cleavage. However, a measure of cell death with appropriately matched controls is not reported in the manuscript. Attributing this observed toxicity to Cas13a requires a robust set of controls that were either not performed or not stated explicitly in the work, including those that address potential toxicity attributed to transfection. To know whether toxicity was related to Cas13a and/or Cas13a-dependent activity would require additionally examining toxicity after transfection of a plasmid encoding a Cas13a only, gRNA only, and ideally a nuclease-deficient Cas13a variant + gRNA. As presented, the conclusion to be drawn is that toxicity is associated with transient transfection after 48 hours.

 In Figures 3 and 4 the authors explore trans cleavage of transcripts in human cells. It should be noted that association of trans-collateral activity with cytotoxicity is only correlative without appropriate controls which demonstrate that toxicity and transcript depletion are both dependent on Cas13a activity. Cells co-transfected with plasmids encoding gRNA and Cas13a were compared to non-transfected controls, which presents a significant difference in treatment of these samples. The authors only have one biological replicate which they attribute to the cellular stress associated with co-transfection, underscoring the possibility of a global shift in transcription as compared to the non-transfected control. Comparing to cells transfected with a nuclease deficient or non-targeting Cas13a, doxycycline only, transfection of vector controls, would help provide evidence that changes in transcript abundance are Cas13-dependent.

 Minor- As the mechanism proposed here is trans-collateral cleavage first requiring target knock down, it would be appropriate to first demonstrate reduction in MALAT and Neo to show that Cas13 is activated, similar to Figure 1E. Was this analysis performed?

 Minor - What was the definition of significant query coverage when aligning gRNA sequences with cDNA from downregulated mRNA? Was a biological justification (seed region, known mismatch allowance) used to support the query coverage and alignment score cutoffs used?

 The main findings illustrated by the authors are that transient transfection and inducible expression of LwaCas13a leads to the most robust expression, and there are detectable differences in transcript abundance after co-transfection. Presentation of the data as such, would positively contribute to the field’s future use of this system.

Is the work clearly and accurately presented and does it cite the current literature?

Partly

If applicable, is the statistical analysis and its interpretation appropriate?

Yes

Are all the source data underlying the results available to ensure full reproducibility?

Yes

Is the study design appropriate and is the work technically sound?

Partly

Are the conclusions drawn adequately supported by the results?

No

Are sufficient details of methods and analysis provided to allow replication by others?

Yes

Reviewer Expertise:

Prokaryotic immunity, CRISPR tool development, RNA editing

I confirm that I have read this submission and believe that I have an appropriate level of expertise to state that I do not consider it to be of an acceptable scientific standard, for reasons outlined above.

# Response

**Published:** 2026-03-04

**Authors:** Elisa Aguilar-Martinez

**Affiliations:** University of Manchester, UK

This work illustrates technical challenges in using and applying LwaCas13a which would be relevant to researchers using this system and potentially other orthologs. However, as stated, the paper lacks the necessary controls to support the conclusions as they are written.

 In Figure 1, the authors suggest that “the cells silenced LwaCas13a because it was either toxic or because incomplete cleavage of the T2A peptide interfered with stability of LwaCas13a.” The concern with the T2A peptide influencing stability is understood, and experiments in Figure 2 convincingly rule this out as the reason for lack of detectable Cas13a expression in Figure 1. Lack of KRAS knockdown supports the lack of robust Cas13a activity under these conditions but does not rule out the possibility that LwaCas13a is simply lowly expressed in these conditions. Given that cells are puromycin resistant and Puro is downstream of Cas13, it is possible that expression is sustained at a level high enough to confer puromycin resistance but not robust Cas13 knockdown. This finding still retains its usefulness to the field—that integration in this context is not conducive to robust knockdown. However, I think that the authors cannot draw a definitive conclusion on this mechanism based on the existing data.

**We thank the referee for the comments. We have modified the text to include this alternative explanation.**

 The authors report LwaCas13a-induced toxicity, consistent with their report of high collateral cleavage. However, a measure of cell death with appropriately matched controls is not reported in the manuscript. Attributing this observed toxicity to Cas13a requires a robust set of controls that were either not performed or not stated explicitly in the work, including those that address potential toxicity attributed to transfection. To know whether toxicity was related to Cas13a and/or Cas13a-dependent activity would require additionally examining toxicity after transfection of a plasmid encoding a Cas13a only, gRNA only, and ideally a nuclease-deficient Cas13a variant + gRNA. As presented, the conclusion to be drawn is that toxicity is associated with transient transfection after 48 hours.

**We have modified the text to make it clearer that transfection of only LwaCas13a (Figure 1B, 1C, 2C), or only gRNA (in cells with undetectable LwaCas13a, 1E) did not lead to cell death,**

 In Figures 3 and 4 the authors explore trans cleavage of transcripts in human cells. It should be noted that association of trans-collateral activity with cytotoxicity is only correlative without appropriate controls which demonstrate that toxicity and transcript depletion are both dependent on Cas13a activity. Please see above comment. Cells co-transfected with plasmids encoding gRNA and Cas13a were compared to non-transfected controls, which presents a significant difference in treatment of these samples. The authors only have one biological replicate which they attribute to the cellular stress associated with co-transfection, underscoring the possibility of a global shift in transcription as compared to the non-transfected control. Comparing to cells transfected with a nuclease deficient or non-targeting Cas13a, doxycycline only, transfection of vector controls, would help provide evidence that changes in transcript abundance are Cas13-dependent.

**We have modified the text to suggest that stress response, not direct cleavage of LwaCas13a could cause the shift in transcription.**

 Minor- As the mechanism proposed here is trans-collateral cleavage first requiring target knock down, it would be appropriate to first demonstrate reduction in MALAT and Neo to show that Cas13 is activated, similar to Figure 1E. Was this analysis performed?

**We have included the RT-qPCR for Neo in figure 2D**

 Minor - What was the definition of significant query coverage when aligning gRNA sequences with cDNA from downregulated mRNA? Was a biological justification (seed region, known mismatch allowance) used to support the query coverage and alignment score cutoffs used? **We defined a “complementary BLAST match with significant query coverage” as any alignment above 20-23 bases and above 90% percent identity threshold.** **These criteria were based on the requirement for activation of Cas13, which needs a near full-length complementarity (allowing one or two mismatches outside the seed region). We have modified the text clarifying this.**

 The main findings illustrated by the authors are that transient transfection and inducible expression of LwaCas13a leads to the most robust expression, and there are detectable differences in transcript abundance after co-transfection. Presentation of the data as such, would positively contribute to the field’s future use of this system.

# Reviewer report: Reviewer response for version 1

**DOI:** 10.21956/wellcomeopenres.26096.r124608

**Published:** 2025-07-02

**Authors:** John C. Thomas

**Affiliations:** 1 University of Cambridge, Cambridge, Cambridge, UK

I think the subject is worthy of discussion and welcome important negative results that can help guide future research away from unproductive areas or at least highlight likely problems other researchers may have if they attempt to use these methods.

 Unfortunately, I find it hard to agree that the provided data and analyses conclusively demonstrate that LwaCas13a has high trans-collateral activity. It's a reasonable conclusion given the lethality of expressing the enzyme. But DGE analysis can only show relative changes in samples as counts must be normalised and can't show unbiased degradation of mRNA in one sample. A bias towards uracil containing transcripts being less abundant would be some evidence. I would also be interested if you see a population of smaller, fragmented mRNA after DOX induction, but I suspect that these may decay quicker and not be found.

 The lack of a definitive statement on the controversy limits the impact of this paper. If further analysis shows evidence of trans-collateral activity that would increase its novelty. However, I think indexing these data and analyses (after revision, see below) would positively contribute to the debate, even without something more definitive.

 I have some comments on the analyses done, and the way the data are presented.

 Fig 2a is very confusing. If it's just supposed to show that mCherry-T2A-LwCas13a is present on one construct, the gRNA-GFP on another construct, and that upon Dox induction you get the 4 proteins produced in the same cell, I'd recommend redrawing it. Having the two constructs to the left of a big arrow labelled "+Dox" and the 4 product proteins to the right of it would be clearer.

 "Of the 62,703 RNAs sequenced, 4,306 and 4,268 were downregulated in gRNA-Neo or gRNA-MALAT1". I'm not sure what "RNAs" means here. Unique transcripts or genes?

 The "differential gene expression analysis" section does not discuss multiple testing correction. DE genes should be defined (at least) as those with an adjusted p-value below a stated threshold. I believe the relevant output from deseq2 is the "padj" column. Table 1 and figure 3 should also report adjusted p-values. And the y-axis line on figure 4 should be defined.

 Though unbiased motif detection makes sense to identify patterns in the down regulated genes, it may make sense to look for a correlation between nucleotide content of the transcripts and their LFC. Assuming the hypothesis that transcripts with more uridines will be cleaved more frequently by Cas13a you may see a difference here. Also note that if the guides are targeting mRNA in an unbiased way, then you would not expect significantly lower expression genes arising from this, as all gene transcripts should be targeted equally, so it doesn't make sense to say that there's an enrichment for genes in essential roles to be down regulated as an explanation for the toxicity. I think it's fine to assert that sufficient random degradation of mRNA would be toxic without showing specific genes being affected.

Is the work clearly and accurately presented and does it cite the current literature?

Partly

If applicable, is the statistical analysis and its interpretation appropriate?

Partly

Are all the source data underlying the results available to ensure full reproducibility?

Yes

Is the study design appropriate and is the work technically sound?

Partly

Are the conclusions drawn adequately supported by the results?

No

Are sufficient details of methods and analysis provided to allow replication by others?

Yes

Reviewer Expertise:

Bioinformatics, computational biology. Some CRISPR biology.

I confirm that I have read this submission and believe that I have an appropriate level of expertise to state that I do not consider it to be of an acceptable scientific standard, for reasons outlined above.

# Response

**Published:** 2025-11-06

**Authors:** Elisa Aguilar-Martinez

**Affiliations:** University of Manchester, UK

I would disagree with the conclusions around figure1 'These results suggest that the cells silenced LwaCas13a because it was either toxic or because incomplete cleavage of the T2A peptide interfered with the translation of LwaCas13a'. Given the construct design in figure 1, I don’t understand how Puro expression could be sustained downstream of the T2A (aka no frame-shift mutation) whilst preventing upstream protein expression. There could maybe be a logic for point mutations in the Cas13a if it were toxic, with Puro expression being maintained but this doesn’t explain the loss of detection of a protein by 3XHA tag detection given I see no reason for point mutations against all three HA tags? Related to this the transient expression of the construct seems to lead to formation of the processed Cas13a product of the expected size (no larger band visible either) suggesting correct processing, so unless there is a reason to expect genomic lentiviral integration to compromise T2A processing I don’t think this explains the result either. I suspect a more probable scenario could be that the expression level from lentiviral integration leads to much lower copy number and reduced expression whereby the sensitivity of the Western Blot is too low to detect but the Puro expression is still high enough to ensure viability of the cells. This would suggest the delivery mechanism/amount may explain the results rather than the protein, in the absence of data suggesting otherwise. Related to the T2A question I don’t see the rationale for testing constructs with T2A both upstream and downstream of the LwaCas13a given the orginal construct seemed to work fine for transient expression of T2A downstream (and enables puromycin survival implying Puro expression assuming the antibiotic is working).    

- *We thank the reviewer for the detailed suggestion. After cleavage, the T2A peptide remains attached to the C-terminal of the upstream protein and previous reports (e.g., Reinhardt, et al.) have shown the T2A peptide can affect protein stability. To explore if this was the case for LwaCa13a, we attached T2A up or downstream of LwaCas13a.  We have modified the text to explain this more clearly. *

 

- *We considered the possibility of low expression of LwaCas13a as a cause for the lack of detection by immunoblot and tested LwaCas13a activity. This was done by transducing the PuroR HEK293T or HaCaT cells with a gRNA targeting KRAS (previously published by Abudayyeh, et. al.) but we did not see knock-down of KRAS. We have included now a new figure showing these data (Figure 1E).  *

      For the Western Blot I believe it is best practice to include loading controls and here it would help rule out some potential reasons for absence of protein expression.

- *We have included a Ponceau stain for the immunoblot for fig 1C and 2C.*

  I didn't think there were appropriate controls to support the conclusion around LwaCas13a toxicity: 'Surprisingly, the expression of LwaCas13a, even at low levels (-Dox), caused significant cell toxicity, with cell death in co-transfected cells within 48 h post-transfection.’ I didn’t see the controls necessary for this conclusion e.g. transduction with only the gRNA lentiviral construct to pinpoint that LwaCas13a delivery was specifically responsible for toxicity observed rather than the transduction delivery process/amounts used. Related to this, viral delivery methods and amounts should be described in the methods section.

- *We thank the reviewer for this comment. We have now modified the text to make clearer how the experiment was carried out. The HEK293T cells were co-transfected with plasmids coding for LwCas13a and gRNA targeting MALAT1 or Neo.*
- *We did not see any toxic effect on the cells when they were transduced. Transduction was used when we first tried to generate the stable cell lines expressing LwaCas13a and also to deliver the gRNA targeting KRAS (Figure 1D) in cells where LwaCas13a expression could not be detected by blot (Figure 1D and 1E). *
- *MOI for the experiments where the cells were transduced are now included in Materials and Methods.*

  For the RNA-seq stage it wasn’t clear to the reader how assessment of significant trans-collateral activity was achieved. There was a description of the numbers of RNAs defined as downregulated between samples for the two comparisons but no benchmarking of the expectations of observing reduced gene expression by chance alone (e.g. comparing between negative control samples). To me this would be necessary to assess whether a significant number of genes showed reduced expression compared to expectations.    

- *The expectation of the experiments was to specifically knock-down MALAT or Neo in the cells transfected with the gRNAs targeting MALAT or Neo. The trans-collateral activity assessment was done by comparing the data from multiple samples LwaCas13a + gRNA -/+ Dox to the un-transfected control. We assessed whether a significant number of genes showed reduced expression compared to expected decrease in MALAT and Neo mRNAs and found 484 mRNAs significantly downregulated in response to LwaCas13a. While we did not have enough negative control samples to do robust statistics on gene expression reduced by chance we compared all of our samples to the untreated cells and found unspecific reduction of mRNAs in all LwaCas13a treated samples. *

* * Assuming the RNA-seq reduction of expression was significant compared to base-line expectations (e.g. WT vs WT) then the over-expression of stress response genes opens up an alternative hypothesis not discussed here that the construct is stressing the cells leading to global shift in transcription. Ideally the authors would address this.

- *While this is a plausible alternative hypothesis, we think that LwaCas13a may have a more specific effect overriding the possible degree of toxicity of transfection. RNA-seq demonstrated a strong reduction of expression in LwaCas13a cells with an enrichment of sequences of the affected mRNAs (Table 1 and Figure 4B). We would have expected even less specific response if the cells were mainly affected by the transfection, which in our protocol was rigorously tested to reduce the effect of the transfection complexes to the minimum. We addressed the stress response observed and showed an increase in the expression of genes involved in cellular stress response (Figure 4C).*

    Minor comments:   In general, the clarity could be raised, e.g. describing the methodological approach / exact experimental setup a little bit more within the results.

- *We have modified the text to explain better the methodology used.*

  Related to the RNA-seq interpretability, if multiple replicates were collected this should be stated as well as the appropriateness of the number of replicates for any statistical analysis performed.

- *Due to the toxic effect of LwaCas13a we could only extract high quality RNA for sequencing from one experiment collecting the cells just prior the maximum toxicity was evident. Therefore, all the analysis was adjusted to parameter that would permit a single replicate. We also “merged” all the samples and compared them against the un-transfected. More detail on the number of replicates and data analysis have been added to the text.*

  When you say in the methods ‘To ensure the robustness of our dataset, genes with zero read counts were excluded and a subset of protein-coding genes was selected.’ Does this mean only protein coding genes were explored or that a subset of protein-coding genes was selected. If the second, then how was selection performed/based on what criteria?

- *Thank you for pointing this out. For the analysis we included the top 200 downregulated coding genes. *

  In the introduction you say, 'In prokaryotic cells, once Cas12 and Cas13 have found and cleaved their specific targets, trans-collateral activity is turned on, resulting in a crRNA-independent, unspecific cleavage of any DNA (Cas12) or RNA (Cas13) present in the cell.’ I think the unspecific cleavage by Cas12 only applies to single stranded DNA not any DNA (e.g. not double stranded DNA).

- *We have modified the introduction to clarify that Cas12 only cleaves single stranded DNA. *

# Reviewer report: Reviewer response for version 1

**DOI:** 10.21956/wellcomeopenres.26096.r125549

**Published:** 2025-07-02

**Authors:** James William Bryson

**Affiliations:** 1 University of Copenhagen, Copenhagen, Denmark

Summarised comments

 Overall, I found the manuscript to be under-developed particularly lacking some key controls. I disagreed with the underpinning conclusions from figure 1. I would also want more clarity around the significance of findings around the RNA-seq data and whether the authors can discount stress response driving transcription changes rather than indiscriminate activity.

 Major concerns:

- In its current form the manuscript needs further development, and I question the value of the first figure panel to the reader especially as I would interpret the conclusions differently (discussed below).
- I would disagree with the conclusions around figure 1 'These results suggest that the cells **silenced LwaCas13a because it was either toxic or because incomplete cleavage of the T2A peptide** interfered with the translation of LwaCas13a'. Given the construct design in figure 1, I don’t understand how Puro expression could be sustained downstream of the T2A (aka no frame-shift mutation) whilst preventing upstream protein expression. There could maybe be a logic for point mutations in the Cas13a if it were toxic, with Puro expression being maintained but this doesn’t explain the loss of detection of a protein by 3XHA tag detection given I see no reason for point mutations against all three HA tags? Related to this the transient expression of the construct seems to lead to formation of the processed Cas13a product of the expected size (no larger band visible either) suggesting correct processing, so unless there is a reason to expect genomic lentiviral integration to compromise T2A processing I don’t think this explains the result either. I suspect a more probable scenario could be that the expression level from lentiviral integration leads to much lower copy number and reduced expression whereby the sensitivity of the Western Blot is too low to detect but the Puro expression is still high enough to ensure viability of the cells. This would suggest the delivery mechanism/amount may explain the results rather than the protein, in the absence of data suggesting otherwise.
- Related to the T2A question I don’t see the rationale for testing constructs with T2A both upstream and downstream of the LwaCas13a given the orginal construct seemed to work fine for transient expression of T2A downstream (and enables puromycin survival implying Puro expression assuming the antibiotic is working).
- For the Western Blot I believe it is best practice to include loading controls and here it would help rule out some potential reasons for absence of protein expression.
- I didn't think there were appropriate controls to support the conclusion around LwaCas13a toxicity: 'Surprisingly, the expression of LwaCas13a, even at low levels (-Dox), caused significant cell toxicity, with cell death in co-transfected cells within 48 h post-transfection.’ I didn’t see the controls necessary for this conclusion e.g. transduction with only the gRNA lentiviral construct to pinpoint that LwaCas13a delivery was specifically responsible for toxicity observed rather than the transduction delivery process/amounts used. Related to this, viral delivery methods and amounts should be described in the methods section.
- For the RNA-seq stage it wasn’t clear to the reader how assessment of significant trans-collateral activity was achieved. There was a description of the numbers of RNAs defined as downregulated between samples for the two comparisons but no benchmarking of the expectations of observing reduced gene expression by chance alone (e.g. comparing between negative control samples). To me this would be necessary to assess whether a significant number of genes showed reduced expression compared to expectations.
- Assuming the RNA-seq reduction of expression was significant compared to base-line expectations (e.g. WT vs WT) then the over-expression of stress response genes opens up an alternative hypothesis not discussed here that the construct is stressing the cells leading to global shift in transcription. Ideally the authors would address this.

Minor comments:

- In general, the clarity could be raised, e.g. describing the methodological approach / exact experimental setup a little bit more within the results.
- Related to the RNA-seq interpretability, if multiple replicates were collected this should be stated as well as the appropriateness of the number of replicates for any statistical analysis performed.
- When you say in the methods ‘To ensure the robustness of our dataset, genes with zero read counts were excluded and a subset of protein-coding genes was selected.’ Does this mean only protein coding genes were explored or that a subset of protein-coding genes was selected. If the second, then how was selection performed/based on what criteria?
- In the introduction you say, 'In prokaryotic cells, once Cas12 and Cas13 have found and cleaved their specific targets, trans-collateral activity is turned on, resulting in a crRNA-independent, unspecific cleavage of any DNA (Cas12) or RNA (Cas13) present in the cell.’ I think the unspecific cleavage by Cas12 only applies to single stranded DNA not any DNA (e.g. not double stranded DNA).

Is the work clearly and accurately presented and does it cite the current literature?

Partly

If applicable, is the statistical analysis and its interpretation appropriate?

Partly

Are all the source data underlying the results available to ensure full reproducibility?

Yes

Is the study design appropriate and is the work technically sound?

No

Are the conclusions drawn adequately supported by the results?

No

Are sufficient details of methods and analysis provided to allow replication by others?

Partly

Reviewer Expertise:

CRISPR tools, sequencing method development, bioinformatics

I confirm that I have read this submission and believe that I have an appropriate level of expertise to state that I do not consider it to be of an acceptable scientific standard, for reasons outlined above.

# Response

**Published:** 2025-11-06

**Authors:** Elisa Aguilar-Martinez

**Affiliations:** University of Manchester, UK

Fig 2a is very confusing. If it's just supposed to show that mCherry-T2A-LwCas13a is present on one construct, the gRNA-GFP on another construct, and that upon Dox induction you get the 4 proteins produced in the same cell, I'd recommend redrawing it. Having the two constructs to the left of a big arrow labelled "+Dox" and the 4 product proteins to the right of it would be clearer.  

- *We apologise that the figure was not clear and thank the reviewer for suggesting how to improve it. Please note that addition of Dox only induces expression of LwCas13a and mCherry. The gRNA and GFP are constitutively expressed. The figure and figure legend have been modified to explain this.*

  "Of the 62,703 RNAs sequenced, 4,306 and 4,268 were downregulated in gRNA-Neo or gRNA-MALAT1". I'm not sure what "RNAs" means here. Unique transcripts or genes?  

- *We use the term RNA to refer to the unique transcripts of genes. The text has been modified accordingly. *

  The "differential gene expression analysis" section does not discuss multiple testing correction. DE genes should be defined (at least) as those with an adjusted p-value below a stated threshold. I believe the relevant output from deseq2 is the "padj" column. Table 1 and figure 3 should also report adjusted p-values. And the y-axis line on figure 4 should be defined.  

- *There are important corrections, thank you for pointing out the missing definitions in the text and on the figures 3 and 4. Due to the intrinsic experimental challenge of extracting good quality RNA from cells undergoing stress, we only have one replicate of the RNAseq data. Therefore, we treated all the samples with a gRNA as “repeats” and compared them against the un-transfected cells. To define the transcripts with significant differential expression, we filter out genes based on read counts and chose the ones with  LFC<-1 and p-adj <0.05. Text, figures and figure legends 3 and 4 have been modified to explain this more clearly.  *

  Though unbiased motif detection makes sense to identify patterns in the down regulated genes, it may make sense to look for a correlation between nucleotide content of the transcripts and their LFC. Assuming the hypothesis that transcripts with more uridines will be cleaved more frequently by Cas13a you may see a difference here. Also note that if the guides are targeting mRNA in an unbiased way, then you would not expect significantly lower expression genes arising from this, as all gene transcripts should be targeted equally, so it doesn't make sense to say that there's an enrichment for genes in essential roles to be down regulated as an explanation for the toxicity. I think it's fine to assert that sufficient random degradation of mRNA would be toxic without showing specific genes being affected.  

- *We explored the possibility that LwaCas13a preferentially degrades transcripts rich on uridines or any other motif. However, discriminative MEME and HOMER analysis did not show this was the case. We have included the results in Figure 4B, confirming that sufficient random degradation of mRNA was toxic.*

- *Indeed, we did not find complementary matches between the gRNAs and the targeted mRNAs. Also, we showed indiscriminate downregulation of genes and mentioned as example genes required for normal cell function of survival. The text has been modified to reflect this better.  *
